# Childhood disability and socio-economic circumstances in low and middle income countries: systematic review

**DOI:** 10.1186/1471-2431-11-119

**Published:** 2011-12-21

**Authors:** Douglas E Simkiss, Clare M Blackburn, Felix O Mukoro, Janet M Read, Nicholas J Spencer

**Affiliations:** 1 Health Sciences Research Institute, Warwick Medical School, University of Warwick, (Gibbet Hill Road), Coventry, (CV4 7AL), UK; 2 School of Health and Social Studies, University of Warwick, (Gibbet Hill Road), Coventry, (CV47 1GN), UK; 3 NHS Kidney Care, New Croft House, (Market Street East), Newcastle upon Tyne, (NE1 6ND), UK; 4 School of Health and Social Studies, University of Warwick, (Gibbet Hill Road), Coventry, (CV47 1GN), UK; 5 School of Health and Social Studies, University of Warwick, (Gibbet Hill Road), Coventry, (CV47 1GN), UK

## Abstract

**Background:**

The majority of children with disability live in low and middle income (LAMI) countries. Although a number of important reviews of childhood disability in LAMI countries have been published, these have not, to our knowledge, addressed the association between childhood disability and the home socio-economic circumstances (SEC). The objective of this study is to establish the current state of knowledge on the SECs of children with disability and their households in LAMI countries through a systematic review and quality assessment of existing research.

**Methods:**

Electronic databases (MEDLINE; EMBASE; PUBMED; Web of Knowledge; PsycInfo; ASSIA; Virtual Health Library; POPLINE; Google scholar) were searched using terms specific to childhood disability and SECs in LAMI countries. Publications from organisations including the World Bank, UNICEF, International Monetary Fund were searched for. Primary studies and reviews from 1990 onwards were included. Studies were assessed for inclusion, categorisation and quality by 2 researchers.

**Results:**

24 primary studies and 13 reviews were identified. Evidence from the available literature on the association between childhood disability and SECs was inconsistent and inconclusive. Potential mechanisms by which poverty and low household SEC may be both a cause and consequence of disability are outlined in the reviews and the qualitative studies. The association of poor SECs with learning disability and behaviour problems was the most consistent finding and these studies had low/medium risk of bias. Where overall disability was the outcome of interest, findings were divergent and many studies had a high/medium risk of bias. Qualitative studies were methodologically weak.

**Conclusions:**

This review indicates that, despite socially and biologically plausible mechanisms underlying the association of low household SEC with childhood disability in LAMI countries, the empirical evidence from quantitative studies is inconsistent and contradictory. There is evidence for a bidirectional association of low household SEC and disability and longitudinal data is needed to clarify the nature of this association.

## Background

In 2004 the Global Burden of Disease report estimated that over 100 million children under the age of 15 years had a moderate or severe disability, the majority of whom live in low and middle income countries (LAMI) [1,2]. Research on these children in LAMI countries however, has been described as 'woefully inadequate' [3]. Reviews of research have found that the majority of studies have focused on cross-sectional, community-based epidemiologic studies aimed at finding the prevalence or aetiology of certain conditions or impairments [3]. In addition, the quality of many studies has been judged inadequate [3-5]. It has been argued that it is important to move beyond prevalence studies to generate informative data on the circumstances of people with disability, including how these compare with those of their non-disabled peers [5]. There is now widespread acceptance that disability definitions and measures should no longer focus solely on impairments and other individual characteristics and conditions, important though those undoubtedly are. Definitions and measures of disability should also incorporate contextual dimensions which may enable or act as barriers to disabled children's participation and human rights [6-9]. A paradigm of disability, which incorporates both individual characteristics and social circumstances, is reflected in the recently developed *International Classification of Functioning Disability and Health - Children and Youth *(ICF-CY) [10] and the *United Nations Convention on the Rights of Persons with Disabilities *(UNCRPD) [11]. These factors make it an imperative to remedy the significant knowledge gap about the lives of children with disability in LAMI countries [1,2,12]. The World Disability Report [1] places emphasis on the role of environmental factors in disability; however, it identifies the absence of clarity on the relationship between household socio-economic circumstances and disability among both children and adults in developing countries. An important starting point is to scope existing studies that address this relationship.

## Objective

A number of important reviews of childhood disability in LAMI countries have already been undertaken and point to significant gaps in knowledge about this important group of children and their families [3,5,13-15]. The purpose of this paper is to establish the current state of knowledge on the socio-economic circumstances (SEC) of disabled children and their households in LAMI countries through a systematic review and quality assessment of existing research. We are taking a broad definition of disability consistent current thinking in the World Report on Disability and for the definition of SEC, we include the following variables; income/asset measures, parental education, poverty, area-based measures of socio-economic factors, occupation-based measures and housing-based measures. To our knowledge, this is the first review specifically reporting the association between childhood disability and the home socio-economic circumstances in LAMI countries.

## Methods

We carried out a review of childhood disability in LAMI countries focusing on four areas: methodological issues in studying childhood disability; household SEC of children with disabilities; the rights of children with disabilities; the use of the International Classification of Functioning, Disability and Health - Children and Young people (ICF-CY) in relation in LAMI countries. This paper is concerned only with the household SEC of children with disability in LAMI countries. Further papers will address the literature related to the rights of children with disability and the use of the ICF-CY. The review by Maulik and Darmstadt [3] covers the methodological issues in detail and our review does not add significantly to their conclusions.

### Search Strategy

#### Databases searched

The search for evidence explored six electronic databases, including medical literature (MEDLINE, EMBASE, and PUBMED), Web of Knowledge and CSA databases (PsycInfo, ASSIA). Broad searches were also conducted in Virtual Health Library, and POPLINE databases, as well as in Google scholar http://scholar.google.co.uk. Publications from organisations such as World Health Organisation (WHO), The World Bank and International Monetary Fund (IMF), United Nations Children's Fund (UNICEF); UN Statistics Division; Statistical Information and Monitoring Programme on Child Labour (SIMPOC); Development Banks (Inter-American; African; Asian); Save the Children; Washington Group; Paris21 consortium (Partnership in Statistics for Development in the 21st. century) and The Foundation for Scientific and Industrial Research (SINTEF) were screened. Bibliographies of selected articles were also searched. We set out to identify all relevant publications regardless of language. Only primary studies and reviews from LAMI countries evaluating SEC of disabled children, methodological issues in childhood disability data collection, the use of ICF-CY, and the rights of children with disability were considered eligible for review. Only literature published between 1990 and June 2009 were included because earlier papers may not now be relevant and the United Nations Convention on the Rights of the Child, the basis of children's rights, was published in 1989.

#### Search terms used

The search terms and strategies used for the searched databases are summarized in Table 1.

**Table 1:** Search terms by databases searched

Database	Search strategy
MEDLINE	exp Disabled Children/or exp Disabled Persons/ exp Sensation Disorders/or exp Learning Disorders/ exp Physical Impairment/ ICF-CY.mp. or international classification of function.mp. exp Disability Evaluation/ child rights.mp. or exp Human Rights/ disability rights.mp. 1 or 2 or 3 or 4 or 5 or 6 or 7 exp Developing Countries/ 8 AND 9 limit 10 to (yr="1990 - 2009" and ("infant (1 to 23 months)" or "preschool child (2 to 5 years)" or "child (6 to 12 years)" or "adolescent (13 to 18 years)"))

EMBASE	exp Handicapped Child/ exp Sensory Dysfunction/ exp Intellectual Impairment/or exp Hearing Impairment/ exp Disability/or exp Physical Disability/or exp Mental Deficiency/ exp Functional Assessment/or exp "International Classification of Functioning, Disability and Health"/or exp Classification/or ICF-CY.mp. exp Human Rights/or child rights.mp. disability rights.mp. 6 or 7 or 4 or 1 or 5 or 3 or 2 exp Developing Country/or low-income countries.mp. 9 and 8 limit 10 to (yr="1990 - 2009" and (infant < to one year > or preschool child <1 to 6 years > or school child <7 to 12 years > or adolescent < 13 to 17 years >))

PUBMED	("developing countries"[MeSH Major Topic]) AND ((("sensation disorders"[MeSH Major Topic]) OR ("mental retardation"[MeSH Major Topic])) OR ("disabled children"[MeSH Major Topic])) Limits: Infant: 1-23 months, Preschool Child: 2-5 years, Child: 6-12 years, Adolescent: 13-18 years

VHL/POPLINE/Google Scholar	("disabled children" OR "disability" OR "ICF-CY") AND ("developing countries")

CSA (PsycInfo, ASSIA)	KW = ((disabled children) or ICF-CY or (disability rights)) or KW = ((sensation disorders) or (sensory impairment) or (mental retardation)) KW = ((developing countries) or (low - income countries) or (poor countries)) or KW = ((latin american countries) or (south east asian countries) or (south american countries)) or KW = ((african south of sahara) or (subsaharan african countries) or (low middle - income countries)) 1 AND 2

#### Inclusion criteria

Both primary studies and reviews relating to household SEC of children with disability in LAMI countries were included. Only quantitative studies with data on the association of childhood disabling conditions and household SEC were included. Seven studies that reported on children and adults combined with no separate analysis of childhood disability and household SEC were excluded. Qualitative studies and reviews were included if they reported on childhood and adult disability and household SEC.

#### Instrumentation

The studies were independently assessed for inclusion and categorization by two researchers, DS and NS. There was a very high level of 96% agreement overall and 92% for included studies, final decisions were made by agreement. All included studies were entered into a spreadsheet and then categorised using variables that included details of the research design, screening tools/method used, primary population studied, specific health condition and broad aim. The key focuses of the papers were categorized according to the broad themes including methodology, household socio-economic circumstances, rights, and ICF/ICF-CY. This paper is concerned only with those papers focusing on the household socioeconomic circumstances of children with disabilities.

### Quality assessment

Papers were assessed for quality using the STROBE criteria http://www.strobe-statement.org modified for the specific requirements of the review (Table 2). Studies were judged to have low risk of bias if they had optimum quality in at least 6 out of the eight quality domains and no domains with least valuable quality. Studies with medium risk of bias were those with less than six optimum quality domains with only one least valuable quality domain or six optimum but with one least valuable domain. High risk of bias was defined as more than one least valuable domain or case series with no controls or comparison group.

**Table 2:** Quality assessment criteria

Quality criterion	Optimum	Adequate	Least valuable
**Design & data collection methods**	Longitudinal	Cross-sectional survey or case-control study	Case series without controls or comparison groups

**Purpose**	Specifically designed to collect childhood disability data	General purpose survey including questions on childhood disability	Inadequate childhood disability data

**Data collection methods**	Interview based	Interview based	Postal questionnaire based

**Quantitative/qualitative**	Combined quantitative & qualitative data on childhood disability	Quantitative only for prevalence estimation; qualitative only to study impact of disability on household	Either quantitative or qualitative data that cannot be adequately used to estimate prevalence or impact

**Definitions & classifications of disability/impairment**	Definition that is widely used, internationally validated & comparable with other data sets	Less than optimal definition but sufficiently detailed to allow reasonable prevalence estimates to be made	Unclear definition that does not allow comparison with other data sets

**Sampling & sample**	Representative of households with children in study country & sufficient size to enable prevalence estimates of less common impairments.	Representative of households with children in study country	Non-representative samples OR Small samples with inadequate numbers to make reliable prevalence estimates

**Response rates**	Full information on response rates with low non-response (< 10%)	Limited information on response rates or non-response (10-20%)	No response information or high response rates (> 20%)

**Household measures of SES & socio-demographic characteristics**	Data covering measures of income, parental education, household wealth & assets in addition to other socio-demographic characteristics including ethnicity, marital status, parental age, parental disability	Limited measures of SES such as maternal education only OR SES measures but limited data on other socio-demographic characteristics	No valid measures of SES or S-D characteristics

**Information on child**	Data on age, sex, school attendance	Limited information on age but no other data	No data on child - only on household as a whole

**Information on children (< 19 years) living outside the home**	Complete data on all children	Limited data on all children	No data on children living outside the home

### Analysis

Summary findings related to the household SEC of disabled children in developing countries were extracted from primary studies and reviews identified by the search. The findings of qualitative studies were summarised. The association of household SEC with childhood disability was expressed as odds ratios with 95% confidence intervals where available or p values where odds ratios were not stated by the authors. Meta-analysis of the findings of the empirical studies was not attempted due to heterogeneity of the studies.

## Results

24 primary studies of the relationship of childhood disability with family and household social circumstances in LAMI countries were identified [2,4,16-38] (see Table 3). We found no reviews focusing specifically on the relationship of childhood disability with family and household social circumstances in LAMI countries. Three reviews [39-41] set out to address the association of both adult and child disability with poverty in LAMI countries; a further 10 reviews [3,5,13-15,42-46] refer to the association within the context of reviewing other aspects of disability in LAMI countries.

**Table 3:** Primary studies of household SEC of children with disabilities

Author/year/country	Study design	Population & sample	Disability measure	Measure of household SEC	Summary of results [Odds ratios (OR) with 95% CIs where available]
**Anselmi et al, 2008** **Brazil^16^**	Prospective cohort study	601 children of 634 randomly selected from the Pelotas birth cohort & followed up between the ages of 4 & 12 years.	Emotional and behavioural problems measured using the Child Behavior Checklist (CBCL) administered at 4 & 12 years of age	Family income	Externalising (OR .72(.52,.96)) and internalising (OR .68(.47,.98)) behaviours and attention problems (OR .57(.39,.84)) at age 12 years were significantly associated with low family income at 4 years

**Bashir et al, 2002 Pakistan^17^**	Prospective cohort study	772 children aged 4-6 years of age out of 1476 births enrolled in a birth cohort in 4 areas of Lahore with contrasting socio-economic characteristics	Mild Mental Retardation (MMR) measured as IQ in the range 50-69 measured using WISC & Griffiths tests among those initially identified using the Ten Question screening test	Four socio-economically distinct areas - village, periurban slum, urban slum & upper middle class area. [no individual or household level SES data reported]	MMR prevalence: Upper middle class 1.4% Village 4.8% Urban slum 6.1% Periurban slum 10.5%

**Bastos et al, 1995** **Tanzania^18^**	Cross-sectional survey	854 children aged 6 to 16 years in schools in the Moshi and Munduli districts of northern Tanzania	Hearing loss measured by pneumotoscopy and screening audiometry	Urban v. Rural areas	Hearing loss in speech frequency range more common in urban (37%) compared with rural (18%) and high frequency loss also more common in urban compared with rural

**Durkin et al, 1998** **Pakistan^19^**	Cross-sectional population survey	6,365 2-9 year old children in Greater Karachi screened in phase 1 of the survey using TQQ; 818 screening positive and 545 of those screening negative assessed in phase 2	Identification of mental retardation by: Phase 1: TQQ screen Phase 2: clinical assessment using Stanford-Binet IQ test & adaptive behaviour scale developed for Pakistani children	Maternal education level (Some v. None) Urban v. Rural	Mild Retardation (IQ 50-70): No education OR 3.08(1.85,6.14) Rural OR 2.33(1.33,2.75) Serious Retardation (IQ < 50): No education OR 3.25(1.86,8.43) Rural OR 2.21(0.87,5.57)

**Filmer 2005** **9 low & middle income countries (Jamaica, Romania, Cambodia, Indonesia, Mozambique, Burundi, Myanmar, Mongolia and Sierra Leone)^20 ^**	11 Nationally representative cross-sectional household surveys in 9 countries - 3 Living Standards Measurement Studies; 3 national socio-economic status surveys; 4 MICS 2 surveys; 1 DHS survey	Children & young people aged 6-17 years - population samples ranged from 1,649 in Jamaica (2000) to 64,136 in Indonesia (2000)	Impairment definitions of disability consistent with ICF's 'body functioning & structure' domain - range of different questions used	Quintiles of Household per capita consumption expenditure in LSMS & SES surveys Quintiles of index of household consumer assets & housing characteristics in DHS & MICS2	Prevalence higher in poorest quintile compared with richest in all countries except Burundi, Cambodia, Mongolia and Mozambique - only Indonesia shows a clear social gradient across quintiles

**Filmer 2008** **13 low & middle income countries** **(Jamaica, Romania, Cambodia, Indonesia, Mozambique, Burundi, Mongolia, South Africa, Chad, India, Colombia, Bolivia and Zambia)^4^** **[NB: some overlap with Filmer 2005]^20 ^**	14 Nationally representative cross-sectional household surveys in 13 countries - 2 Living Standards Measurement Studies; 5 national socio-economic status surveys; 2 MICS 2 surveys; 5 DHS surveys	Children & young people aged 6-17 years - population samples ranged from 5,865 in Burundi (2000) to 140,297 in India (1992)	Impairment definitions of disability consistent with ICF's 'body functioning & structure' domain - range of different questions used	Quintiles of Household per capita consumption expenditure in LSMS & SES surveys Quintiles of index of household consumer assets & housing characteristics in DHS & MICS2	Only Indonesia & India show clear differences in disability prevalence between poorest & richest quintiles - otherwise non-significant differences

**Grut & Ingstad, 2006** **Yemen^21^**	Qualitative study	28 interviews involving 38 individuals in households with disabled people, & one group interview in an institution for disabled girls [15 children; 20 adults; 2 Disabled People's Organisations]	Range of disabilities including physical, intellectual, hearing & visual [interviews of those with intellectual impairments conducted with parents in presence of the disabled person]	Specific measures not used but study does examine the impact of disability on the lives of households with disabled members particularly children and explores the association of poverty & disability	Complex relationship between disability and poverty demonstrated with insights into how disability and poverty interact to limit life chances

**Hackett et al, 1999** **India^22^**	Random cluster sampling for cross-sectional survey	1326 children aged 8-12 years in 2 local government districts outside the city of Calicut, Kerala State, India	Child psychiatric disorder identified in 2 phases: Phase 1: Screening interviews using various instruments including Rutter's A2 scale plus Rutter teacher completed B2 scale Phase 2: detailed psychiatric assessment of screen positive & 93 screen negative	Poverty index based on eight household characteristics Father's occupation in 5 categories from professional to unskilled Parental education level - age ceased formal education	Externalising behaviours: associated with low occupation group, low parental education & poverty Internalising behaviours: associated with low parental education

**Ingstad & Grut, 2007** **Kenya^23^**	Qualitative study	42 interviews (27 individual; 4 group; 11 secondary information) including 16 children in 7 strategically chosen districts of Kenya	Range of disabilities including physical (33), intellectual/mental (9), hearing (8) & visual (5)	Specific measures not used but study does examine the impact of disability on the lives of households with disabled members particularly children and explores the association of poverty & disability	Complex relationship between disability and poverty demonstrated with insights into how disability and poverty interact to limit life chances

**Kandamuthan, 1997** **India^24^**	Case-control study nested within Health and Disability cross-sectional survey of 9652 households in 1983 in an area of Trivandrum, Kerala State.	180 children aged 0-14 years with identified disability compared with 900 controls.	Questionnaire & clinical assessment: 8 outcomes studied: total disabled; fits; speech & hearing disability; visual impairment; learning disability; strange behaviour; locomotor disability; other	20 SES measures used but not fully stated in the paper. Following univariate analysis, maternal education, family size and absence of latrine retained in multi-variate analysis	Total disability associated with: Low maternal education - adjusted OR 2.46 (1.03,5.89); Family size > 5 - adjusted OR 3.71(2.44,5.63); have latrine - adjusted OR 0.59(0.41,0.84)

**Kuklina, 2006** **Guatemala^25^**	Cross-sectional study nested within a birth cohort including all pregnancies between 1996 & 1999	385 children at 6 months of age; 342 at 24 months of age and 404 at 36 months of age in 4 villages in Eastern Guatemala	Neurodevelopment measured using the Mental Development and the Psychomotor Development indices of the Bailey Scales of Infant Development	Maternal education and household SES based on household characteristics and possessions	Maternal education & SES were not associated with child neurodevelopment

**Loaiza & Cappa, 2005** **7 low/middle income countries^26^**	Cross-sectional MICS2 surveys in Cameroon, Iraq, Jamaica, Lesotho, Madagascar, Sao Tome & Principe, and Suriname during the period 1999-2001	Children 2-9 years - sample sizes not stated	TQQ	Rural v. Urban Maternal education Wealth quintiles	Disability prevalence - tends to be higher in rural areas although varies by country; tends to be higher among less educated mothers but variable; variable relationship with wealth index - Suriname & Madagascar show social gradients in the expected direction but no significant differences in the other 5 countries

**Meeks Gardner et al, 1995 & 1999** **Jamaica^27, 28^** **[2 papers combined as same study described]**	Nested randomised control trial within a larger study	78 stunted children & 26 non-stunted children in poor neighbourhoods of Kingston Jamaica - data collected at 12 & 24 months of age	Mental age measured using the Griffiths Global Development scores	Housing quality - measured by quality of sanitation & water supply and overcrowding Caldwell HOME inventory - quality of home environment Maternal score on Peabody Picture Vocabulary Test (PPVT) of verbal reasoning	Mental age at 12 & 24 months associated with maternal PPVT Not associated with housing quality or HOME score

**Mung'ala-Odera et al, 2006** **Kenya^29^**	Cross-sectional survey	10218 out of 11416 children aged 6-9 years in the Kilifi district of Kenya (one of the poorest districts in the country - mainly subsistence farmers)	Neurological impairment measured in 2 phases: Phase 1 - TQQ screen Phase 2 - psychological & neurological assessment undertaken by trained researchers among all screen +ve & similar number of screen -ve children	Maternal education Mother not involved in economic activity Father not involved in economic activity	Moderate/severe impairment NOT associated with any of the SES measures on univariate analysis

**Natale et al, 1992** **India^30^**	Cross-sectional survey	640 children aged 2-9 in households randomly sampled from 2 urban areas of Madurai, Tamil Nadu with contrasting socio-economic characteristics	TQQ screen with additional probe questions to ensure that only chronic conditions identified. TQQ responses grouped into 4 subscales - sensory; neuromotor; cognitive; verbal	Residence in one of 2 urban areas: one a slum area with residents of the lowest SES (monthly family income 10-15 US$/month); the other a slightly higher SES area (monthly family income 32-42 US$)	Overall disability: 17.4% in lowest SES area v. 8.2% in next lowest SES area In logistic regression model adjusted for age, gender, birth order & number in family OR for lowest 2.39(1.82,3.09) All subtypes except verbal significantly higher in lowest SES area

**Rischewski, 2008** **Rwanda^31^**	Nationwide matched case-control study (adults & children) nested within a national cross-sectional survey	93 cases aged < 15 yrs identified in the national survey matched for age & gender with 146 controls	Musculoskeletal impairment (MSI) ranging from knock knees to quadriplegia	Per capita household expenditure; x2 household asset possession measures	MSI in children < 15 NOT associated any of the household poverty measures

**Shawky, 2002** **Saudi Arabia^32^**	Case-control study based on four cross-sectional cohorts	1225 children aged 6-20 years with disabilities identified from specialist centres in Jeddah and 3405 non-disabled school children sampled from 42 boys' and 42 girls' schools	Auditory, visual & mental impairment	Maternal education; maternal working status	No maternal education associated with: auditory impairment OR 13.3 (7.2,27.8); visual impairment OR 3.7 (2.1,6.6); mental impairment OR 5.5 (3.8,8.1) - all adjusted for maternal age at birth, parity, working status, consanguinity & multiparity

**Suris & Blum, 1993** **> 20 countries - high & low income^33^**	Secondary data analysis of cross-sectional surveys	10-14 yr olds in 19 countries and 15-19 yr olds in 23 countries - only rates calculated - no numbers given. Data derived from UN International Disability Statistics Database (DISTAT)	No specific definition of disability stated - total disability rates as reported to UN	% female illiteracy	% country level female illiteracy not correlated with disability rates for adolescents in either age group

**Izutsu et al, 2006** **Bangladesh^34^**	Cross-sectional survey	Random samples of 187 boys & 137 girls from non-slum areas and 157 boys and 121 girls from slum areas of Dhaka aged 11-18 years	Mental health problems (Affective, anxiety, somatic, oppositional defiant & conduct problems plus ADHD) assessed using the Youth Self Report questionnaire administered in their homes by trained interviewers	Residence in slum or non-slum areas	Only conduct problems associated with living in slum areas OR 3.2(1.4,7.2) adjusted for gender, age & school enrolment

**Thomas, 2005** **Cambodia^35^**	Qualitative study	Key informant interviews at 3 centres for disabled persons in Cambodia - interviews with staff and administrators; focus group interviews - one with 13 disabled adults & one with 4 disabled children; home visits and interviews with four disabled adults and four disabled children & their parents	Range of different disabilities	Poverty	Poverty identified as both cause and consequence of disability - discussion of mechanisms by which poverty impacts on disability and vice versa based on qualitative data

**Thomas, 2005** **India^36^**	Qualitative study	Field visits to 7 centres for disabled persons. Focus group interviews with 27 at one centre & 12 at another centre. Small number of individual interviews with disabled persons or parents of disabled children	Range of different disabilities	Poverty	Poverty identified as both cause and consequence of disability - discussion of mechanisms by which poverty impacts on disability and vice versa based on qualitative data

**Thomas, 2005** **Rwanda^37^**	Qualitative study	Key informant interviews at 3 centres for disabled persons in Rwanda - 2 focus group interviews (27 and 20 disabled persons) and 4 individual interviews. No specific reference to interviews with children or parents of children.	Range of different disabilities	Poverty	Poverty identified as both cause and consequence of disability - not specific to children

**UNICEF, 2008** **18 low/middle income countries^2^**	MICS3 cross-sectional surveys in Albania, Bangladesh, Belize, Bosnia and Herzegovina, Cameroon, Central African Republic, Georgia, Ghana, Mauritania, Mongolia, Montenegro, Sao Tome & Principe, Serbia, Sierra Leone, Suriname, Thailand, TYFR Macedonia and Uzbekistan during the period 2005-2008	Children aged 2-9 years ranging from 1,537 children in Belize to 58,441 in Bangladesh	TQQ screening	Household wealth index (60% poorest v. 40% richest) Maternal education Rural/urban	Wealth index: only in 6 countries (Bangladesh, Georgia, Mongolia, Serbia, Sierra Leone & Thailand were disabled children at greater risk of living in the poorest 60% of households Low maternal education: only 6 countries show a greater risk for disabled children of living in households with mothers with low education Rural v. Urban: very little association of disability with rural living

**VanLeit et al, 2007** **Cambodia^38^**	Cross-sectional survey	500 children 0-18 years with disabilities - no controls included in the study	Full range of disabilities - objective was to identify the functional status of disabled children in Cambodia	Poverty (< 1$/day)	49% of households identified in the survey were living in poverty

Of the identified primary studies, 5 reported qualitative data within country level reports and 19 were quantitative studies conducted in more than 50 LAMI countries (Table 3). Five studies reported multi-country data, one of which [33] included developed countries as well as developing countries. The age range of children included in the quantitative studies varied from 0-20 years; eight studies reported on children under the age of 10 years, 2 on adolescents only and the remaining studies included young children as well as adolescents. The qualitative data included both adults and children; however, the ages of the children were not specified.

Two of the quantitative studies were based on prospective cohort studies [16,17] and one (reported in two papers) on a randomised control trial [27,28]; the remaining 16 were cross-sectional surveys or case-control studies (Table 3). A range of disabilities were studied in the quantitative studies; total disability rates were reported in 8 studies, emotional & behavioural disorders and mental retardation in 6, hearing loss in 2, visual impairment in 1, and neurological, neurodevelopment and musculoskeletal impairment in 4. The most commonly used instrument to measure disability in the quantitative surveys was the Ten Questions Questionnaire (TQQ); this was used in 8 studies, four as a screening tool in an initial phase of the study and four as the main measure of disability. The qualitative data were based on interviews and focus group including adults and children (or their parents) with a range of disabilities including hearing, visual, intellectual and motor impairment.

Eleven quantitative studies used multiple measures of household SEC. Parental education (mostly maternal education) and income/asset-based measures were the most commonly used measures. Six studies used area-based measures, two occupation-based measures and two housing-related measures. The poverty measures used in the reports containing qualitative data were not specified.

Although the reviews that primarily address disability and poverty [39-41] categorically state that poverty is both a cause and consequence of disability, they review very limited empirical data on the relationship and none related specifically to the household SEC of children with disability in LAMI countries. As shown in Table 3 the qualitative data describe a close association of disability (both adult and child) with poverty; however, the association is not reported consistently in the quantitative studies. Potential mechanisms by which poverty and low household SEC may be both a cause and consequence of disability are outlined in the reviews and the qualitative studies. The reviews identified the living conditions of poor people in LAMI countries as a primary causal mechanism. Preventable impairments associated with communicable, maternal and perinatal disease and injuries [40], and malnutrition in childhood [39] were identified as key mechanisms by which poverty caused disability. The reviews (see particularly Mitra [41]) presented socially plausible mechanisms by which disability itself both in adults and children causes poverty and exacerbates existing poverty. Baylies [43] and Dudzik *et al *[44] identified social structures, war and social unrest which have the greatest impact on the poor as mechanisms linking poverty and disability. Smith [46] reviewing hearing impairment in developing countries, identified poor preventive health services as a factor in high prevalence of deafness and hearing impairment among children due to perinatal factors, chronic otitis media and ototoxic drugs.

The qualitative data provided further support for the role of poverty as both cause and consequence of disability. Ingstad and Grut [23], based on case studies derived from their fieldwork in Kenya, identified congenital conditions, conditions occurring in pregnancy and childbirth, malaria and epilepsy as mechanisms through which poverty leads to disability. All five reports contained rich data from their case studies illustrating how disability exacerbates and precipitates poverty.

Of the 8 studies reporting total childhood disability rates by household SEC, three were single country studies [24,30,38]. In Kandamuthan's case-control study [24] disabled children aged 0-14 years were more than twice as likely to have a mother with low education as their non-disabled peers. Natale *et al's *study [30] reported a more than twofold increase in prevalence of disability (17.4%) among children aged 2-9 years among those living in the lowest socioeconomic area compared with children living in a slightly more advantaged but still poor area (8.2%). Although VanLeit *et al *[38] report 49% of the children aged 0-18 years in their study living in poverty, their study has no control group so the significance of this finding is difficult to interpret. By contrast, there is no consistent association of total childhood disability with household SEC reported by the five multi-country studies [2,4,30,26,33]. Six out of the nine countries included in Filmer's study [20] show significant difference in prevalence of childhood disability by household SEC although only one, Indonesia, shows a clear social gradient. However, in the same author's 2008 study [4], in only two out of 13 countries, Indonesia and India, were significant differences between rich and poor households found. The studies by Loaiza & Cappa [26] and UNICEF [2], based on the Multi-Indicator Cluster Surveys 2 (MICS2) and 3 (MICS3) respectively, report variable findings with only a few countries showing significant relationships of total disability among 2-9 year old children with poorer household SEC. In their study, Suris and Blum [33] use United Nations disability data from both high and low/middle income countries to examine the association of country-level prevalence of disability among adolescents aged 15-19 years. They found no correlation of country level female illiteracy with prevalence of disability in this age group.

The six studies that examined the association of child mental illness, behavioural problems and mental retardation with household SEC [16,17,19,22,32,34] all reported significant relationships with poor household SEC. Bashir *et al *[17], Durkin *et al *[19] and Shawky [32] reported a strong association of mild mental retardation with household SEC. Durkin *et al *[19] reported a strong association of severe mental retardation (IQ < 50) with no maternal education and rural living. Anselmi *et al *[16], in a prospective cohort study, found a significant association of externalising and internalising behaviours, and attention problems at age 12 years with low family income at 4 years of age. Hackett *et al *[22] reported similar findings in a cross-sectional survey. Izutsu *et al *[34] found a significant association of residence in a slum area with conduct problems but not with other behavioural difficulties.

None of the studies reporting on the association of neurological, neurodevelopmental and musculoskeletal problems with household SEC [25,27-29,31] found any significant association. Shawky [32] in a case-control study, found a very high odds ratio (13.3(95% CI 7.2,27.8)) of children and young people aged 6-20 years with hearing loss having a mother with no education. Bastos *et al *[18] reported a two-fold increase in prevalence of hearing loss within the speech frequency range in urban areas (37%) compared with rural areas (18%) of northern Tanzania. The only study reporting on visual impairment [32] found a significant association with no maternal education.

The quality of included studies was variable. Table 4 summarises the quality assessment of the quantitative studies using the criteria listed in Table 2. Seventeen of the 19 quantitative studies had a medium or low risk of bias. The main sources of potential bias were single cross-sectional survey design and high or unreported non-response rates. Two studies had high risk of bias: Suris and Blum [33] as the study was based on national disability rates with no information on definitions, no information of how data were collected and only national level female illiteracy rates as a measure of socioeconomic status; VanLeit *et al *[38] as the study included only adolescents with disabilities with no comparison or control group.

**Table 4:** Quality assessment of quantitative studies

Study	Design	Purpose	Data collection methods	Sampling & sample size	Definitions	Response rate	Measures of home circumstance &/or SES	Child data	Overall risk of bias
**Anselmi et al, 2008^16^** **Brazil**	Optimum	Adequate	Optimum	Optimum	Optimum	Optimum	Adequate	Optimum	Low

**Bashir et al, 2002** **Pakistan^17^**	Optimum	Optimum	Optimum	Optimum	Optimum	Adequate	Adequate	Optimum	Low

**Bastos et al, 1995** **Tanzania^18^**	Adequate	Optimum	Optimum	Optimum	Optimum	Optimum	Least valuable (urban v. Rural)	Optimum	Medium

**Durkin et al, 1998** **Pakistan^19^**	Adequate	Optimum	Optimum	Optimum	Optimum	Optimum	Adequate	Optimum	Low

**Filmer 2005** **9 low & middle income countries^20 ^**	Adequate	Adequate	Optimum	Optimum	Optimum	Least valuable	Adequate	Optimum	Medium

**Filmer 2008** **13 low & middle income countries^4^** **[NB: some overlap with Filmer 2005]^20^**	Adequate	Adequate	Optimum	Optimum	Optimum	Least valuable	Adequate	Optimum	Medium

**Hackett et al, 1999** **India^22^**	Adequate	Optimum	Optimum	Optimum	Optimum	Adequate	Optimum	Optimum	Low

**Kandamuthan, 1997** **India^24^**	Adequate	Optimum	Optimum	Optimum	Optimum	Adequate	Optimum	Optimum	Low

**Kuklina, 2006** **Guatemala^25^**	Adequate	Optimum	Optimum	Adequate	Optimum	Adequate	Optimum	Optimum	Medium

**Loaiza & Cappa, 2005** **7 low/middle income countries^26^**	Adequate	Adequate	Optimum	Optimum	Optimum	Least valuable	Optimum	Optimum	Medium

**Meeks Gardner et al, 1995 & 1999** **Jamaica^27, 28^** **[2 papers combined as same study described]**	Adequate	Adequate	Optimum	Adequate	Optimum	Optimum	Adequate	Optimum	Medium

**Mung'ala-Odera et al, 2006** **Kenya^29^**	Adequate	Optimum	Optimum	Optimum	Optimum	Adequate	Adequate	Optimum	Medium

**Natale et al, 1992** **India^30^**	Adequate	Optimum	Optimum	Adequate	Optimum	Optimum	Least valuable	Optimum	Medium

**Rischewski, 2008** **Rwanda^31^**	Adequate	Optimum	Optimum	Adequate	Adequate	Optimum	Adequate	Optimum	Low

**Shawky, 2002** **Saudi Arabia^32^**	Adequate	Optimum	Optimum	Optimum	Optimum	Optimum	Adequate	Optimum	Low

**Suris & Blum, 1993** **> 20 countries - high & low income^33^**	Least valuable	Optimum	Adequate	Least valuable - only national rates given	Least valuable - no information on definitions used	Least valuable	Least valuable - only national level of female illiteracy given	Least valuable	High

**Izutsu al, 2006** **Bangladesh^34^**	Adequate	Optimum	Optimum	Adequate	Optimum	Optimum	Least valuable	Optimum	Medium

**UNICEF, 2008** **18 low/middle income countries^2^**	Adequate	Adequate	Optimum	Optimum	Optimum	Least valuable	Adequate	Optimum	Medium

**VanLeit et al, 2007** **Cambodia^38^**	Least valuable	Optimum	Adequate	Adequate	Optimum	Optimum	Adequate	Optimum	High

The methodologies used in the country-based reports to collect qualitative data, while enabling rich data to be collected, had weaknesses related to inadequately described sampling methods and limited explanation of how themes and case studies were identified, selected and analysed. The samples included both adults and children limiting the validity of the findings for studying childhood disability.

## Discussion

To our knowledge, this is the first review specifically reporting on the available literature on the association of childhood disability with home socio-economic circumstances in LAMI countries. We identified primary quantitative and qualitative studies and reviews that specifically addressed the household SEC of disabled children in LAMI countries. As this is a narrative review, we have not carried out meta-analysis of results of empirical quantitative studies.

### Main findings

This review has shown that evidence from available literature on the association of childhood disability and household SEC in LAMI countries is inconsistent and inconclusive. This is likely to be due to differences in: measures of household SEC used in the studies; definitions of disability; outcomes studied; study design and methods. Some measures of household SEC, for example, residence in urban versus rural areas and female illiteracy rates are likely to be less precise than those based on individual household SEC measures. Studies using more than one measure of household SEC are more likely to identify associations with childhood disability than those using single measures as different measures may capture different pathways and mechanisms associated with the outcomes. For example, mild learning disability (termed mental retardation in the included studies) is likely to be associated with household education level while hearing impairment is likely to be associated with poor perinatal care and lack of preventive health care secondary to family poverty.

The definition of disability has developed in recent decades and the International Classification of Functioning, Disability and Health promotes a bio-psycho-social model that incorporates components of the medical and social models [10]. Disability arises out of the interaction between health conditions with contextual factors including the environment (SEC is part of this) and personal factors. With the United Nations Convention on the Rights of Persons with Disability, disability is becoming a rights based issue and its definition is changing [11]. However the literature described in this paper defines disability in terms of impairments.

The association of poor household SEC with learning disability and behaviour problems was the most consistent finding of the review. All six studies reported a positive association of poor household SEC with these outcomes and all these studies had a low or medium risk of bias. By contrast, studies with neurodevelopmental and neuromuscular problems as the outcome reported no association with poor household SEC. These studies also carried a low or medium risk of bias. One possible explanation for this differential association is that children with physical or neurological problems die prematurely and so are not available for counting whereas children with learning disability or behavioural problems survive; another explanation is that the neurodevelopmental and neuromuscular conditions may be more likely to have a genetic origin not associated with SEC.

Only two studies reported on hearing loss as the outcome of interest. They reported strikingly different associations with SEC. These divergent findings may be partly explained by the different measures of household SEC used.

The studies in which overall disability was the outcome of interest also reported divergent findings. The multi-country studies, based on secondary analysis of the UNICEF Multiple Indicator Cluster Surveys (MICS), Demographic and Health Surveys and other survey datasets, reported a positive association of childhood disability rates with poor household SEC in a few countries but no association in the majority of countries studied. Children with disability were identified using the TTQ in several of these studies. This instrument was designed with two stages; the questionnaire and then a clinical assessment of children who scored positively on the questionnaire. Unfortunately the clinical assessment is often not performed, making the TQQ invalid. One of these studies [33] had a high risk of bias; the other four studies had a medium risk of bias. A positive association of childhood disability rates with poor household SEC was reported by the three studies that collected and analysed primary data; however, one of these studies [38] had a high risk of bias as it was based on a case series with no control or comparison group.

The reviews identified by the search strategy reported socially and biologically plausible mechanisms by which poverty might be both a cause and consequence of disability. They identified the living conditions of poor people in LAMI countries as a primary causal mechanism; as Elwan [40] states "disability in developing countries stems largely from preventable impairments associated with communicable, maternal and perinatal disease and injuries". Malnutrition was identified as a specific cause of disability in childhood [39]. The reviews (see particularly Mitra [41]) presented socially plausible mechanisms by which disability causes poverty and exacerbates existing poverty.

The qualitative data from country-based reports had methodological weaknesses that limited the validity of their findings in relation to the association of poor household SEC with childhood disability. However, the studies provided further support for the role of poverty as both cause and consequence of disability. Ingstad and Grut [23], based on case studies derived from their fieldwork in Kenya, identified congenital conditions, conditions occurring in pregnancy and childbirth, malaria and epilepsy as mechanisms through which poverty leads to disability. All five studies reported rich data from their case studies illustrating how disability exacerbates and precipitates poverty.

### Comparison with literature from high income countries and other literature from LAMI countries

To our knowledge, no equivalent review of the association of childhood disability with poor home circumstances/low SES in high income countries has been published. There is a substantial literature from the UK (for example, Gordon *et al *[47]; Blackburn *et al *2010 [48], Emerson *et al *[49]), the USA (for example, Newacheck [50]; Newacheck and Halfon [51]) Australia (for example, Bor *et al *[52]; Leonard *et al *[53]) and Scandinavia (for example, Hjern *et al *[54]; Berntsson and Kohler [55]) showing that childhood disability is associated with higher risk of living in a poor/low income household. Gordon *et al *[47] assert that poverty in high income countries is both cause and consequence of childhood disability but we are not aware of specific studies confirming that poverty is causally related to disability in childhood.

The findings of this review are consistent with the literature in high income countries in that poverty is viewed as both cause and consequence of childhood disability; however, although there is strong evidence in both high income and LAMI countries for poverty as a consequence of disability, there are no empirical studies demonstrating the causal relationship of poverty to disability. Whereas studies from high income countries show a consistent association of childhood disability with poor home circumstances/low SES, the quantitative studies reviewed here show a much less consistent association. This is likely to result from differences in definition of disability, in data quality and sampling methods.

Three quantitative studies [56-58] not included in this review as they did not analyse child and adult disability separately, reported lower incomes and fewer assets among households with disabled members (adults and/or children) and those with no disabled members in three sub-Saharan African countries (Namibia, Malawi and Zimbabwe). Their findings indicate that the use of rigorous research methodology with attention to sampling, disability definition, and detailed measurement of household SEC can result in very similar findings in different countries.

### Limitations

Publication bias is a possible threat to the conclusions of this review. Although we aimed to identify a broad range of journal papers and reports on childhood disability, we did not include a search of grey literature, hand-searching of key journals or advice from key informants. As a consequence, the review is unlikely to include all available studies of the links between household SEC and childhood disability in LAMI countries. We used general search terms for disability, did not include specific diagnoses and chose to limit the search to publications from 1990 onwards in order to exclude studies with outdated information and to limit the volume of literature identified. This may have led to significant omissions from the review.

### Implications for further study

The reviews and qualitative studies included in this review suggest that poverty is both a cause and consequence of disability in childhood. They outline biologically and socially plausible mechanisms by which poverty may exert its influence. By contrast, the empirical studies reviewed here report an inconsistent association of household SEC with childhood disability. Further research in this area is essential to clarify the relationship. The findings of this review, and those of Maulik and Darmstadt [3] indicate the need to address the following issues in future research into the association of childhood disability with household SEC:

**Definitions of disability **- although many of the studies reviewed here clearly stated the definitions used, there is a need, as Maulik and Darmstadt [3] point out, for consistency and standardisation of the definitions used and awareness of the problems of definition that impose limitations on conclusions that can be drawn

**Types of disability **- this review is consistent with Maulik and Darmstadt's [3] observation that the majority of studies focus on intellectual impairment and overall disability rates. Future studies could focus on other relatively common disabling conditions such as cerebral palsy. We found no studies using functional definitions of disability, such as defined in the ICF-CY - these would provide further valuable insights into the association with household SEC

**Measures of household SEC **- the review demonstrates that more than one measure of household SEC is necessary to study comprehensively the association with childhood disability as different measures such as maternal education and household wealth may have different associations with disability.

**Study design **- cross-sectional surveys, the most common design among the quantitative studies reviewed here, are relatively cheap to organise and are valuable for descriptive epidemiology. Further study to describe the relationship of childhood disability and household SEC will be valuable and cross-sectional surveys will continue to have a role. However, such surveys are only able to show associations; longitudinal prospective studies are necessary to comment on causal mechanisms particularly as relates to poverty as a cause rather than consequence of disability.

**Mixed methods studies **- the review shows that the qualitative studies provide valuable insights into possible social and biological mechanisms by which poverty might impact on disability. Future research would be strengthened by a mixed methods design that combined rigorous qualitative research preferably nested within a well-designed prospective quantitative study.

## Conclusion

This review indicates that, despite socially and biologically plausible mechanisms underlying the association of low household SEC with childhood disability in LAMI countries, the empirical evidence from quantitative studies is inconsistent and contradictory. There is an urgent need for a more robust evidence base to inform the development of effective health and social policies aimed at reducing the burden of childhood disability in LAMI countries.

## List of abbreviations

ICF-CY: International Classification of Functioning, Disability and Health - Children and Young People; IMF: International Monetary Fund; MICS: Multiple Indicator Cluster Survey; LAMI: Low and middle income; Paris21: Partnerships in Statistics for Development in the 21^st ^century; POPLINE: Population information online; SEC: socio-economic circumstances; SIMPOC: Statistical Information and Monitoring Programme on Child Labour; SINTEF: The Foundation for Scientific and Industrial Research; TQQ: Ten Questions Questionnaire; UNCRPD: United Nations Convention on the Rights of Persons with Disabilities; UNICEF: United Nations Children's Fund; WHO: World Health Organization.

## Competing interests

The authors declare that they have no competing interests.

## Authors' contributions

DS critically appraised the papers and helped to draft the manuscript. CB conceived of the study and participated in its design and coordination. FM conducted the literature search. JR conceived of the study and participated in its design and coordination NS critically appraised the papers and drafted the manuscript. All authors read and approved the final manuscript.

## Pre-publication history

The pre-publication history for this paper can be accessed here:

http://www.biomedcentral.com/1471-2431/11/119/prepub
